# LLM-Powered Prediction of Hyperglycemia and Discovery of Behavioral Treatment Pathways from Wearables and Diet

**DOI:** 10.3390/s25175372

**Published:** 2025-08-31

**Authors:** Abdullah Mamun, Asiful Arefeen, Susan B. Racette, Dorothy D. Sears, Corrie M. Whisner, Matthew P. Buman, Hassan Ghasemzadeh

**Affiliations:** 1 College of Health Solutions, Arizona State University, Phoenix, AZ 85054, USA; aarefeen@asu.edu (A.A.); susan.racette@asu.edu (S.B.R.); dorothy.sears@asu.edu (D.D.S.); cwhisner@asu.edu (C.M.W.); mbuman@asu.edu (M.P.B.); 2 School of Computing and Augmented Intelligence, Arizona State University, Tempe, AZ 85281, USA

**Keywords:** machine learning, metabolic health, continuous glucose monitoring, diabetes, hyperglycemia, large language models

## Abstract

Postprandial hyperglycemia, marked by the blood glucose level exceeding the normal range after consuming a meal, is a critical indicator of progression toward type 2 diabetes in people with prediabetes and in healthy individuals. A key metric for understanding blood glucose dynamics after eating is the postprandial Area Under the Curve (AUC). Predicting postprandial AUC in advance based on a person’s lifestyle factors, such as diet and physical activity level, and explaining the factors that affect postprandial blood glucose could allow an individual to adjust their behavioral choices accordingly to maintain normal glucose levels. In this work, we develop an explainable machine learning solution, GlucoLens, that takes sensor-driven inputs and utilizes advanced data processing, large language models, and trainable machine learning models to estimate postprandial AUC and predict hyperglycemia from diet, physical activity, and recent glucose patterns. We use data obtained using wearables in a five-week clinical trial of 10 adults who worked full-time to develop and evaluate the proposed computational model that integrates wearable sensing, multimodal data, and machine learning. Our machine learning model takes multimodal data from wearable activity and glucose monitoring sensors, along with food and work logs, and provides an interpretable prediction of the postprandial glucose patterns. GlucoLens achieves a normalized root mean squared error (NRMSE) of 0.123 in its best configuration. On average, the proposed technology provides a 16% better predictive performance compared to the comparison models. Additionally, our technique predicts hyperglycemia with an accuracy of 79% and an F1 score of 0.749 and recommends different treatment options to help avoid hyperglycemia through diverse counterfactual explanations. With systematic experiments and discussion supported by established prior research, we show that our method is generalizable and consistent with clinical understanding.

## 1. Introduction

Wearable sensors have become indispensable tools in the field of healthcare and personalized medicine, offering continuous, non-invasive monitoring of physiological parameters in real time [1,2]. Devices such as continuous glucose monitors (CGMs), physical activity trackers, and smartphone sensors provide valuable insights into an individual’s metabolic health, physical activity, and lifestyle habits [3,4]. These sensors enable the collection of granular data that can be used to understand complex physiological processes, predict health outcomes, and design interventions [5,6]. Wearable sensors also provide opportunities for developing robust solutions despite different challenges, such as small dataset, missing features, data in the wild, etc. [7,8,9]. In metabolic health, wearable sensors allow for the precise tracking of blood glucose fluctuations and physical activity levels, offering individuals and clinicians the means to implement timely and tailored strategies for disease prevention and management [10]. This accessibility to continuous health data marks a significant shift toward proactive and data-driven healthcare solutions.

Hyperglycemia, or high glucose concentration in the blood, occurs when the body cannot effectively regulate glucose levels. Hyperglycemia in the fasted state (i.e., no caloric intake for at least 8 h) is defined by a fasting plasma glucose level of ≥100 mg/dL (≥5.6 mmol/L), whereas hyperglycemia in the non-fasted state generally is defined as having a blood glucose level (BGL) of ≥140 mg/dL (7.8 mmol/L) two hours after a meal [11,12]. Lack of physical activity and relative overconsumption of carbohydrates are known to affect a person’s metabolism and their ability to regulate glucose [13]. Other common risk factors often cited as potentially responsible for developing diabetes are being obese, overweight, or having a higher than normal body mass index (BMI) [14]. Untreated hyperglycemia increases the risk of complications like retinopathy, nephropathy, neuropathy, cardiovascular disease, stroke, poor limb circulation, and depression [15]. While hyperglycemia can affect anyone, individuals with prediabetes or diabetes are at a higher risk of developing complications arising from frequent exposure to hyperglycemia, compared to healthy people [16,17].

**Figure 1:**
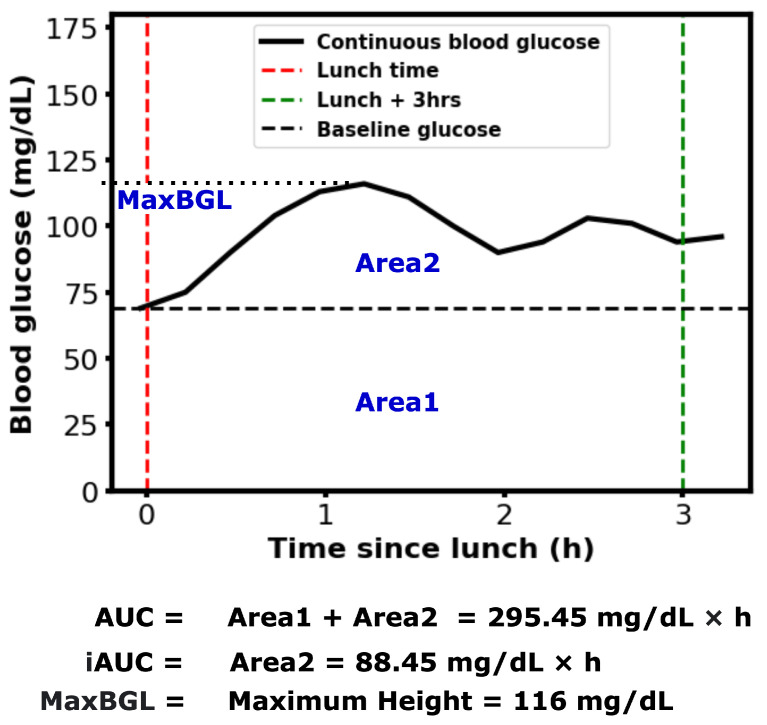
An example of postprandial Area Under the Curve (AUC), incremental AUC (iAUC), and Maximum postprandial Blood Glucose Level (MaxBGL) for one participant after a lunch meal.

The Centers for Disease Control (CDC) estimates that 38% of American adults have prediabetes and 19% of them are unaware of their condition [18]. To help prevent the increasing prevalence of hyperglycemia and prediabetes, the Food and Drug Administration (FDA) has approved the sale of Continuous Glucose Monitors (CGMs) over the counter in the U.S. in 2024 [19]. This decision has made CGM devices more accessible to people with or without diabetes. Prediabetes can be reversed with proper lifestyle management, such as diet and physical activity [20]. However, if left untreated, it can develop type 2 diabetes, which is an irreversible lifelong condition [21]. Healthy individuals are generally expected to maintain their blood glucose levels (BGLs) within a range of 60 to 140 mg/dL [11]. However, temporary elevations above this range are common following certain meals. The area under the BGL curve during a specific post-meal period (e.g., 2–3 h) is referred to as the postprandial Area Under the Curve (AUC). Postprandial blood glucose and AUC are important indicators of BGL regulation and diabetes complications [22,23]. Numerous studies have explored methods for calculating AUC and the various factors influencing it [24,25,26]. An example of calculating AUC, incremental AUC, and the maximum postprandial blood glucose level (MaxBGL) is shown in Figure 1.

Blood glucose forecasting using wearable sensor data is an active area of research that has been addressed with different machine learning and deep learning approaches, including ensemble methods, attention methods, and knowledge distillation [27,28]. While the area under the glucose curve has gained attention as a promising metric for estimating hyperglycemia risk [29,30], existing research has not yet explored how comprehensive lifestyle factors, including diet, physical activity, work routines, and baseline glucose parameters, can be integrated to predict both hyperglycemia and glucose curve characteristics.

In this paper, we present GlucoLens, a machine learning framework that integrates continuous glucose monitoring (CGM) data, activity tracker measurements, and food and work logs to estimate postprandial area under the glucose curve (AUC) and predict hyperglycemia. To summarize, our contributions are as follows: (i) designing hyperglycemia and AUC prediction systems based on machine learning and large language models that combine wearable data, food logs, work logs, and recent CGM patterns; (ii) implementing GlucoLens for AUC and hyperglycemia prediction and diverse counterfactual (CF) explanations; (iii) training and testing our models on a dataset collected in a clinical trial conducted by our team; (iv) evaluating the explanations using objective metrics and by comparing them with the prior clinical research, and (v) providing a thorough discussion of our findings and how it advances the research of hyperglycemia prediction. Code available: https://github.com/ab9mamun/GlucoLens (accessed on 29 June 2025).

## 2. Materials and Methods

### 2.1. System Overview

**Figure 2:**
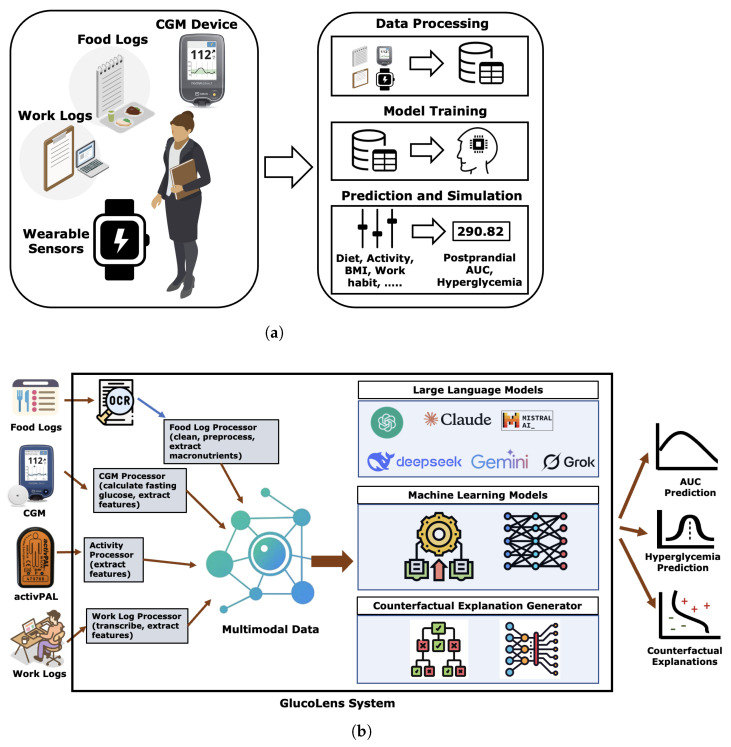
The GlucoLens system’s body sensor network is composed of food logs, work logs, a CGM device, and a wearable sensor. Data from all these sources are combined to create a unified dataset to train a model. The machine learning models are further supported by large language models (LLMs) and data augmentation tools based on Gaussian noise. Finally, counterfactual explanations are provided so that interventions can be applied. (**a**) Simplified workflow of the GlucoLens system. (**b**) Detailed pipeline of the GlucoLens system.

To investigate the questions about the relationships of diet and activity with postprandial BGL, we designed a system of four data sources: a wearable device with motion sensors, a CGM device, food logs, and work logs. The system’s data processing tools process the raw data from multiple sources and convert them to compatible electronic formats. The processed data are then used to train ML models, which are capable of making accurate predictions about the AUC and hyperglycemia given the type of diet and activity. The system is illustrated in Figure 2.

**Figure 3:**
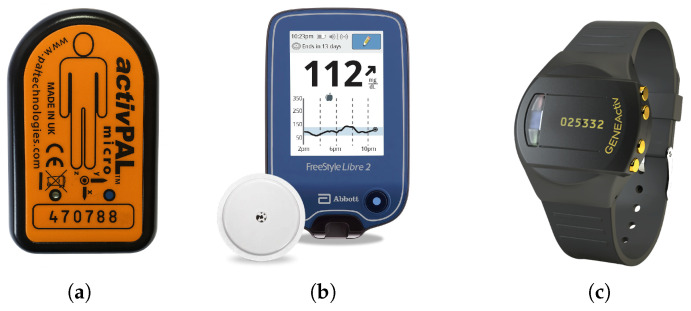
Wearables with sensors in the WorkWell Study. activPAL uses a tri-axial accelerometer for detecting activities. The FreeStyle Libre CGM patch measures glucose levels in the interstitial fluid (the fluid surrounding cells) using a thin filament inserted under the skin. The GENEActiv device uses a 3-axis accelerometer, a light sensor (photodiode), and a temperature sensor. (**a**) activPAL micro device, to be worn on thigh. (**b**) Freestyle Libre CGM patch and reader. (**c**) GENEActiv wearable wristband.

The system has three wearable devices: a FreeStyle Libre CGM device (Abbott Laboratories, Abbot Park, IL, USA) an activPAL micro device (PAL Technologies, Glasgow, Scotland, UK), and a GENEActiv wearable wristband (ActivInsights, Kimbolton, UK), as shown in Figure 3. activPAL provides event data with the timestamps of that specific event [31]. Changes in activity are considered as events by the activPAL device, e.g., when a person stands up from a sedentary position or starts moving from a standing position. The different activities or events detected by the device are sedentary, standing, stepping, cycling, primary lying, secondary lying, and seated transport. For each event, the event start time and the duration of the event are recorded. Duration spent in different activities can be calculated from those events. The Freestyle Libre provides CGM readings in 15 min intervals. GENEActiv provides various biomarkers including acceleration, physical activity intensity, sedentary vs movement activity, and sleep–wake time [32].

GlucoLens is composed of a multimodal data processing package, interfaces for an array of large language models (LLMs), trained machine learning models curated for postprandial AUC and hyperglycemia predictions, and a counterfactual explanation generator. The data processing package includes Optical Character Recognition (OCR), food log processor, CGM processor, activity processor, and work log processor. Our system processes the raw data from the sensors and these sources and prepares a multimodal dataset. The multimodal data is used to produce predictions from 7 different LLMs: GPT-3.5-Turbo, GPT-4, Claude Opus 4, Deepseek V3, Gemini Flash 2.0, Grok 3, and Mistral Large. The Mistral Large model declined to provide predictions and was thus omitted from the results and not considered for the hybrid predictors. Additionally, machine learning backbones such as Random Forest (RF), Ridge Regressor, Multilayer Perceptron (MLP) Regressor, as well as XGBoost [33], and TabNet [34]. The multimodal dataset is augmented by adding small Gaussian noise to the scaled features. The system then produces predictions for postprandial AUC, maximum blood glucose level, and postprandial hyperglycemia. Finally, diverse counterfactual explanations are provided so that interventions can be designed to avoid hyperglycemia.

### 2.2. Wearables and Lifestyle Data

**Figure 4:**
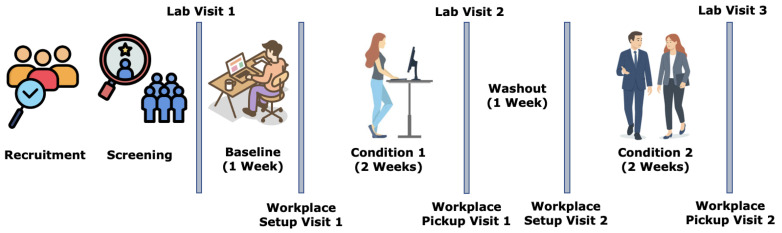
Timeline of the WorkWell Study. Each participant completed a 1-week Baseline phase data collection, 2 weeks of Condition 1 phase data collection, and 2 weeks of Condition 2 phase data collection. There was a 1-week break between Condition 1 and Condition 2. In all three phases, participants provided CGM, activPAL, GENEActiv, food logs, and work logs data. The Baseline phase was the control phase, where the participants maintained their usual work habits. Participants were randomly assigned an intervention of ‘Stand’ or ‘Move’ in Condition 1, and the other intervention was assigned in Condition 2. In the ‘Stand’ intervention, they were prompted to stand 6 minutes more per hour at work than their Baseline measured mean, and during the ‘Move’ intervention, they were prompted to move 6 minutes more per hour at work than their Baseline measured mean.

To develop the proposed system, we used a subset of data obtained in the WorkWell Study [35], a clinical trial (NCT04269070) conducted by our team that involved adults who worked full time. The WorkWell Study was approved by the Arizona State University Institutional Review Board, and all participants provided written informed consent to participate. Each participant was given a CGM device and an activPAL wearable [36] device. Lunch was delivered at work from select restaurants on every working day (five days per week) for one work week at Baseline, two work weeks during Condition 1, and two work weeks during Condition 2, as shown in Figure 4. Each participant received printed forms to maintain the food logs and work logs. The option of homemade meals was not considered for lunch on working days because it would make the task of estimating the macronutrients difficult. The selected restaurants already had the macronutrients for all their items available online, which was used to estimate the nutritional components for every meal consumed.

In the food logs, the participants noted down the time and what they ate in every meal, including if there were any leftovers. The participants also logged the amount of water and any supplements (e.g., multivitamins) they were taking every day. In the work logs, they noted when they started working, when they stopped working, how they arrived at work or if they were working from home, and approximately what percentage of working time they spent sitting, standing, and walking. The Baseline week did not have any intervention, but the participants wore their CGM devices and activPAL. Two interventions for Condition 1 and Condition 2 were ‘recommending to be standing’ and ‘recommending to be moving’ for 6 minutes longer per hour than the corresponding mean values at the Baseline phase. All participants were provided with both interventions, but the temporal order was random. Therefore, for some participants, the temporal order was the Baseline, Stand, Move, and for the other participants, it was the Baseline, Move, Stand. In this study, we have included our analysis based on the data of 10 participants having an average Baseline BMI of 32.8±4.5.

### 2.3. Data Processing

In this study, one of the main interests was predicting postprandial blood glucose during work days and investigating how diet and activity during work days affect postprandial AUC. Moreover, the participants were provided standardized lunch on working days, whereas they ate anything they liked on weekends. In the food logs, the participants kept track of any leftover portions, which allowed us to accurately extract the amount of calories and macronutrients consumed during lunch. The food logs were maintained as handwritten logs. They were processed through Google Cloud Vision OCR to create electronic logs, followed by some human intervention for issues that could not be resolved by the OCR. The amounts of macronutrients consumed were used to estimate glycemic loads (GLs) using the formula from [37], as shown in Equation (1).

(1) $$\begin{array}{cc} GL=19.27 & +(0.39\times \mathrm{net}\;\mathrm{carb}.)-(0.21\times \mathrm{fat})\\ & -\left(0.01\times {\mathrm{protein}}^{2}\right)-\left(0.01\times {\mathrm{fiber}}^{2}\right) \end{array}$$

The work logs were also handwritten, and they were processed manually to convert them to an electronic format. From activPAL sensors data and with the help of work logs, the durations of sitting, standing, and stepping were calculated for the day to lunch, as well as the durations of sitting, standing, and stepping during work hours for the day until lunch. For this work, fasting glucose was defined as the minimum CGM reading between 6 AM and 10 AM. The recent CGM was defined as the average CGM reading of the same day from 12 AM to 8 AM. A complete list of the input features of the different feature sets that were formed can be found in Table 1. The ‘Sensor+Macro’ feature set uses activity metrics from the sensor data and calculated macronutrients, along with the common features that are present in all feature sets. Two additional inputs are used in the ‘All’ feature set: an activity score calculated from self-reported activity logs during work, and the glycemic load. In the ‘Self+Macro’ feature set, the 6 features containing sitting, standing, and stepping durations are replaced by the activity score based on self-reported activity logs. The activity score was calculated from the self-reported activities in the work logs. In the work logs, each participant reported the percentage of their working hours spent sitting, standing, and walking. The recent activity score is calculated by taking the average percentage of time spent in walking activity in the previous days of the same phase and adding with $\frac{1}{2}\times$ the average percentage of time spent in standing activity in the previous days of the same phase. In the ‘Self+GL’ and ‘Sensor+GL’ feature sets, the macronutrients, including net carbs, fat, protein, and fiber, are removed and replaced with the glycemic load calculated in Equation (1). The relationship of all five different feature sets can be found in Table 1.

### 2.4. ML Models

Five different architectures, i.e., Random Forest (RF), Ridge Regressor, MLP Regressor, XGBoost, and TabNet, were trained as potential backbone architectures of the GlucoLens system. MLPs and Random Forests are capable of making effective predictions with proper feature extractions [38], whereas linear regression methods with regularization, such as Ridge Regression [39] and Lasso Regression [40], are popular choices as comparison models. Neural networks are usually data-hungry, which is why, on small datasets, classical ML methods can show competitive and even better performance than neural networks.

**Figure 5:**
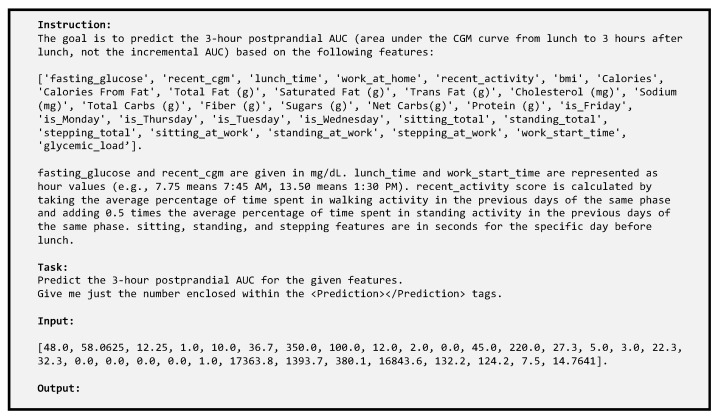
A sample zero-shot prompt for the LLMs. The input field is varied for each different example; everything else in the prompt remains the same.

Additionally, zero-shot large language models were used for LLM-only predictions based on the feature values. The structure of the prompt used for the LLMs is presented in Figure 5. The content for the “Input:” field is changed for each different datapoint, whereas the rest of the body of the prompt remains the same. In the next step, hybrid predictors that use both zero-shot LLM predictions and a trainable regressor (RF or XGBoost) were implemented. Three variations in the hybrid predictors were implemented and trained. The first variation (Gly_Hybrid) uses the existing features and adds the predictions by six advanced LLMs as additional inputs. The second hybrid variation (Gly_Hybrid_v2) uses the existing features and the prediction by only the best-performing LLM (in our case, Claude Opus 4) for training and testing. Finally, the third hybrid variation (Gly_Max) uses a Gaussian noise-based augmented feature set based on existing features, predictions by Claude Opus 4, and a trainable regressor. Three variations in RF, three variations in Ridge, thirteen variations in MLP, one version of XGBoost, and one version of TabNet were trained and tested for the Gly_Base version of the GlucoLens system. The different models and variations for AUC, MaxBGL, and Hyperglycemia prediction are presented in Table 2 and Table 3. For AUC and MaxBGL predictions, 20% of data instances were assigned to the test set, and 80% of data instances were assigned to the training set. A total of 159 datapoints were used for training. Out of them, 127 were used for training and 32 were used for testing. For hyperglycemia detection, six different variations in the train–test split were used, which are discussed in detail in Section 3.4.

### 2.5. Hyperglycemia Detection and Counterfactual Explanations

**Figure 6:**
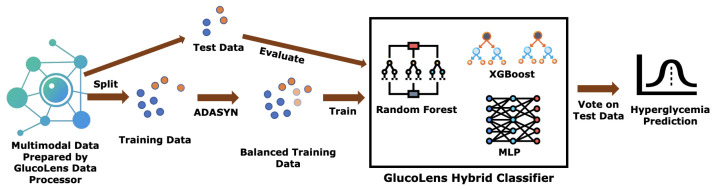
The pipeline of classification of hyperglycemia with GlucoLens system’s hybrid classifier. A soft voting based on the probabilities of classes suggested by the RF, XGBoost, and MLP (version 13 of Table 3) is used to make the final predictions. This hybrid method outperforms the prediction performances of single classifiers. The test set contains only real datapoints, so that the evaluation is not biased by the data generation method, whereas the balanced training data contains both real and synthetic datapoints.

GlucoLens was also trained for hyperglycemia detection with RF, XGB, MLP, backbone models, as well as hybrid combinations of these three, as shown in Figure 6. More details about all the different variations in the models and different splits of training and test sets are described in Section 3.4. Counterfactual explanations can provide insights into features responsible for an undesired health outcome and possible remedies to overcome them [41]. As a primary goal of the paper was to explore more knowledge about the reasons for and ways to prevent hyperglycemia, a DiCE-based counterfactual explanations generator is integrated with GlucoLens. GlucoLens provides multiple counterfactual explanations that are diverse and achievable with small perturbations from the original example [42].

## 3. Results

### 3.1. Prediction of AUC with Different Backbone Architectures

The first problem we want to solve is the prediction of postprandial AUC and we used five different architectures as the backbone of our model, including 3 different versions of Random Forest (RF), 3 different versions of Ridge regressors, 13 versions of MLP, and finally, XGBoost [33] and TabNet [34]. According to our experiments, GlucoLens performs the best with an RF backbone with a normalized root mean squared error (NRMSE) of 0.123. TabNet trained with 100 epochs provided an NRMSE of 0.147, and XGBoost provided an NRMSE of 0.137. A summary of the performance of all these different backbones can be seen in Table 4. We believe that advanced neural networks integrated with our system will be able to produce more accurate results, but it will require more data to train them. We show in Section 3.4, where the results of hyperglycemia detection are discussed, that the performance on the test set monotonically improves as we increase the size of the training dataset.

### 3.2. Prediction with LLMs

**Figure 7:**
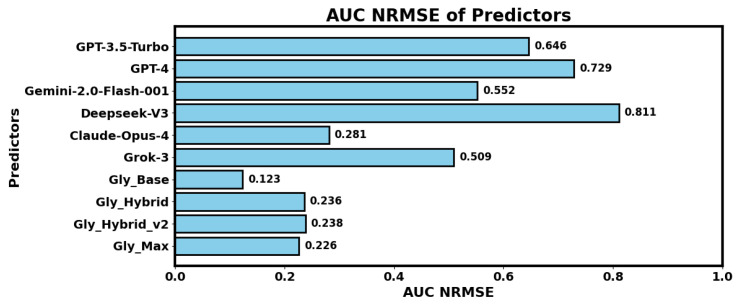
Normalized root mean squared errors on the prediction of AUC with different LLMs and hybrid classifiers of our system. Explanations of Gly_Base and the hybrid predictors can be found in Table 2. Explanations of the feature sets can be found in Table 1. ANOVA [43] test for LLM-based predictions (F = 210.13, *p* < 0.001). Significance thresholds are F > 1 and *p*-value < 0.05.

The results presented in Table 5 and Figure 7 showcase the performance of various configurations of our proposed solution. These configurations include Gly_Base, which does not utilize large language models (LLMs); Gly_LLM, a zero-shot prediction approach using only LLMs; Gly_Hybrid, combining LLM-based predictions with our system; Gly_Hybrid_v2, which integrates predictions exclusively from the best-performing LLM (Claude); and Gly_Max, an enhanced version of Gly_Hybrid_v2 with augmented training data incorporating Gaussian noise. Performance of the hybrid models was evaluated by integrating LLMs with one of the two best-performing regressor backbones: Random Forest (RF) or XGBoost. Among the configurations, Gly_Base with RF backbone achieved the best performance. Interesting findings can be observed in Figure 7, where we can see that Claude Opus 4 outperformed all other LLM-based models by a large margin. These findings indicate that it may not be wise to ask LLMs for personalized meal plans as they cannot accurately predict the consequences of a certain combination of diet and other factors on the postprandial blood glucose levels. We used the ‘All’ feature set for the LLM-based experiments.

### 3.3. Performance of MLP and Prediction of MaxBGL

**Figure 8:**
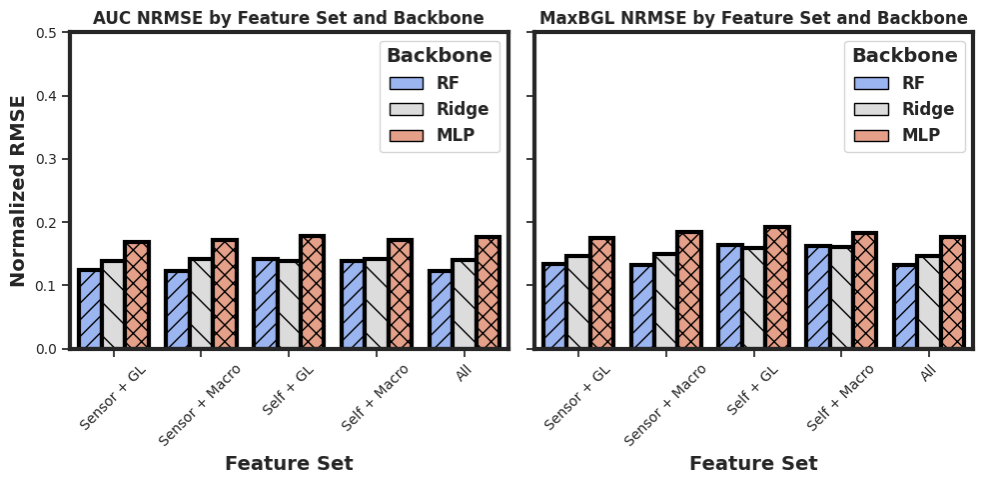
Summary of the best results for each target outcome with RF, Ridge, and MLP regressors. Three different models were tested with five different combinations. Meanings of the outcomes AUC and MaxBGL can be found in Figure 1. RF = Random Forest. NRMSE = Normalized Root Mean Squared Error. ANOVA [43] test for AUC predictions (F = 73.84, *p* < 0.001) and for MaxBGL predictions (F = 16.52, *p* < 0.001). Significance thresholds are F > 1 and *p*-value < 0.05.

Two different outcomes were chosen for the regression problem of predicting AUC and MaxBGL. In Figure 8, we see that for AUC and MaxBGL, the best NRMSE values are 0.123 and 0.132, respectively. RF predictors obtained all three best results. Although 13 different variations were tested for MLPs, none of them achieved the best performance on any of the three tasks.

**Figure 9:**
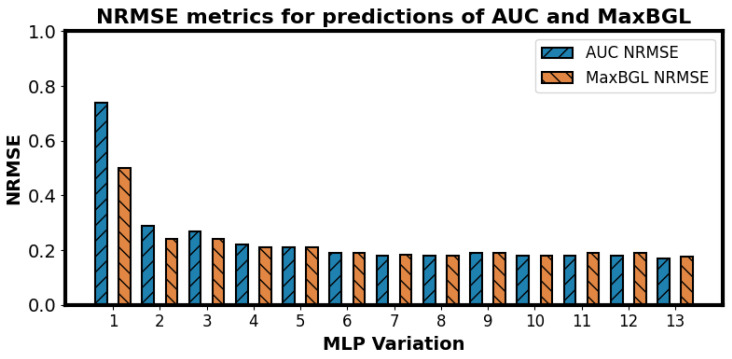
Normalized root mean squared errors (NRMSE) for different variations in MLP in two different regression tasks. Best NRMSE for AUC prediction is 0.169, and best NRMSE for MaxBGL prediction is 0.175. The details of the MLP variations can be found in Table 3. The best performance among MLPs was achieved with the MLP variation no. 13 and the feature set ‘Sensor + GL’.

Out of the 13 different variations in MLP regressors, all of their NRMSE metrics were higher than the corresponding RF regressors, as shown in Figure 8. It encouraged us to look deeper into the results of the MLP variations. We present the best performances by each of the 13 MLP variants for both outcomes in Figure 9. MLP variation no. 13 is the largest of all the MLP variations used in our experiments, and it performed better than any other MLP versions in both tasks. It raises the question of whether even larger and deeper models would be more accurate and potentially better than the RF and Ridge models. We wish to investigate this in future work.

### 3.4. Results of Hyperglycemia Detection

The results for different classifiers can be seen in Table 6. We notice that the hybrid classifier that uses soft voting among an ensemble of RF, XGB, and MLP outperforms the other combinations of classifiers. Note that the performance improvement in ensemble classifiers over individual classifiers is not due to the increased number of parameters because each model of the ensemble classifiers is trained independently (on the same training dataset), and only during inference on the test dataset, they work together to make more reliable predictions. We used the ‘All’ feature set for the hyperglycemia detection experiments.

RF, XGB, MLP, and their combinations were first trained and evaluated for a split of approximately 87% training and 13% test set. This split was created by randomly separating 10 positive class (hyperglycemia) examples and 10 negative class (normal) examples to create the test set. The rest of the examples were used to create the training set. Thus, the test set was already balanced, and the training set was balanced with ADASYN [44].

In the next part of the experiments, we show that as we increase the size of the training set, the performance keeps increasing. We varied the size of the training set from 70% of the total dataset to up to 99% of the dataset, and we noticed a strictly increasing pattern in all four metrics until the training set is 95% of the dataset, as seen in Table 7. This supports the fact that with a bigger dataset, the performance of our method can be much higher than what we are getting with this small dataset. For the last case, when 99% of the dataset is used for training, the test set consists of only 1 example from each class. This case makes the evaluation of the test set highly vulnerable for the precision and recall metrics, as unless the classifier predicts the whole test set perfectly in any trial, one of these two metrics would be zero for that trial, which would affect the F1 score as well. However, we believe that in a significantly larger dataset, even 1% of the dataset could be used for testing. Overall, the best performance was found with RF+XGB+MLP with 95%, 5% train–test split with an accuracy of 73.3% and a macro average F1 score of 0.716 with a precision of 0.751 and a recall of 0.733.

GlucoLens achieves an accuracy of 79% and an F1 score of 0.749 in hyperglycemia detection with a small dataset of 159 data points, as shown in Table 8. Furthermore, we prove with experiments that the performance improves as we increase the size of the dataset, as seen in Table 7.

### 3.5. Impact of Augmentation and Evaluation on Unbalanced Test Set

Table 8 compares the performance of the hybrid RF+XGB+MLP model with and without data augmentation using metrics such as accuracy, precision, recall, and F1 score. Across three evaluation runs with 10-fold cross-validation, the use of augmentation consistently improved performance. On average, accuracy increased from 0.763 to 0.790, and similar gains were observed in precision (from 0.723 to 0.756), recall (from 0.725 to 0.745), and F1 score (from 0.724 to 0.749). These improvements suggest that data augmentation helped the model generalize better, especially in the context of an unbalanced test set. The results highlight the benefit of augmentation strategies for enhancing robustness and predictive reliability in clinical applications.

### 3.6. Further Tests for Generalizability—Leave-One-Subject-Out and Leave-One-Out Experiments

Table 9 presents the results of the leave-one-subject-out evaluation for assessing model generalizability under different experimental variations. Every variation was repeated for 20 trials, and the average values of 20 trials were used for reporting the performance metrics. Without personalization or augmentation, the model achieved an accuracy of 0.600 and an F1 score of 0.568, reflecting baseline performance when no domain adaptation strategies were applied. Introducing training data augmentation improved accuracy to 0.630 and precision to 0.601, suggesting that synthetic data generation enhanced feature diversity. Personalization, achieved by moving two samples (one positive and one negative) from the test set to the training set without data leakage, further increased performance to 0.648 accuracy and 0.611 F1 score. The best results were obtained when combining personalization with training data augmentation, yielding 0.690 accuracy, 0.654 precision, and 0.651 recall, highlighting the complementary benefits of both techniques in improving generalization to unseen subjects.

Table 10 presents the results of the leave-one-out analysis across three trials. The hybrid RF+XGB+MLP model achieved accuracy values between 0.755 and 0.786, with precision, recall, and F1 score following a similar stable pattern. The average performance across trials was 0.772 accuracy, 0.733 precision, 0.739 recall, and 0.735 F1 score. These results demonstrate the model’s robustness under leave-one-out conditions, indicating consistent predictive behavior even when nearly the entire dataset is used for training while leaving out one sample at a time.

### 3.7. Interventions with Diverse Counterfactual Explanations

**Figure 10:**
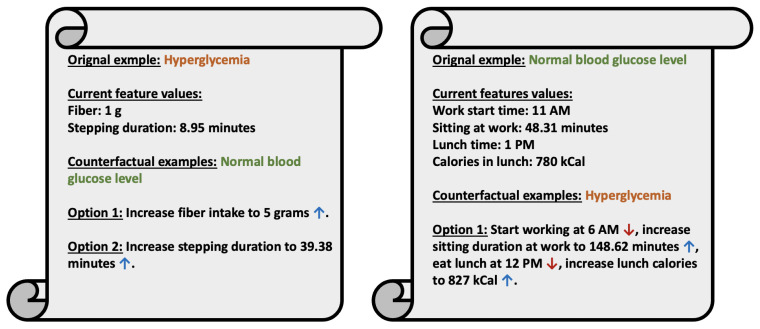
Counterfactual explanations for a normal and a postprandial hyperglycemic event. Features not presented in the figures had the same value in the original and all its counterfactual examples. In the left subfigure, we present two alternative counterfactuals for avoiding hyperglycemia. A blue upward arrow next to a feature value indicates that the value has increased from the original feature value, whereas a red downward arrow indicates that the value for that feature has decreased from the original value. These counterfactuals are consistent with clinical understanding, as increasing fiber intake slows down gastric emptying and glucose absorption in the bloodstream [45]. Similarly, increasing physical activity also helps lower blood glucose levels [46]. In the right subfigure, we present a scenario where a normal BGL could lead to hyperglycemia. The objective of providing the second example is to show what things need to be avoided. According to the counterfactual in the right subfigure, increasing sitting duration and lunch calorie intake can increase the likelihood of hyperglycemia, which is consistent with clinical understanding [47,48]. While the timing of starting work day and lunch time may not directly affect hyperglycemia, we believe they might be correlated and influence other important factors, such as breakfast. Hyperglycemia can be caused by the second meal effect, for example, not having a proper breakfast, which could lead to a much higher BGL spike after lunch [49].

The visualization of counterfactual explanations in Figure 10 highlights insights for avoiding hyperglycemia by examining the influence of various features on postprandial blood glucose outcomes. The first example of Figure 10 represents a hyperglycemic case, characterized by a fiber intake of 1 g and a stepping duration of 8.95 min. Counterfactual scenarios suggest pathways to achieve normal blood glucose levels. Option 1 illustrates that increasing fiber intake to 5 g can lead to a normal outcome. Option 2 shows that substantially increasing stepping duration to 39.38 min also results in a normal blood glucose level. These scenarios emphasize the impact of dietary and physical activity modifications in managing glucose levels.

In contrast, a second example in Figure 10 represents a normal blood glucose level with current feature values of a work start time of 11 PM, sitting duration of 48.31 min, lunch time at 1 PM, and a lunch calorie intake of 780 kCal. Counterfactual scenarios reveal potential transitions to hyperglycemia. The counterfactual example generated against that example demonstrates how starting work earlier at 6 AM, increasing sitting duration to 148.62 min, shifting lunch to 12 PM, and increasing lunch calories to 827 kCal can contribute to hyperglycemia. These findings highlight the importance of balancing work habits, meal timing, and physical activity for glucose management, underscoring the value of counterfactual explanations for personalized recommendations.

### 3.8. Evaluation and Clinical Validity of Counterfactual Explanations

Table 11 presents the evaluation metrics for the generated counterfactual examples. The counterfactuals achieved perfect validity (1.000), indicating that all generated instances successfully flipped the prediction. An average diversity score of 3.945 suggests that the generated examples are meaningfully distinct from one another, helping prevent redundancy. The normalized distance of 2.258 reflects the degree of change from the original instances while maintaining plausibility. Finally, an average of two features changed per counterfactual indicates a balance between interpretability and effectiveness.

The evaluation metrics also suggest strong potential for clinical validity. The perfect validity score (1.000) indicates that each counterfactual leads to a definitive and reliable change in prediction, which is essential for clinical decision support. The relatively low number of features changed (2.0 on average) enhances interpretability, making the counterfactuals easier for clinicians to understand and act upon. Moreover, the moderate normalized distance (2.258) and high diversity (3.945) imply that the counterfactuals are both realistic and varied, capturing multiple plausible paths to improved outcomes. Together, these properties make the generated explanations clinically meaningful and actionable in real-world settings. Moreover, the counterfactuals generated are consistent with the clinical understanding, as explained in Figure 10.

### 3.9. Explanation with SHAP

**Figure 11:**
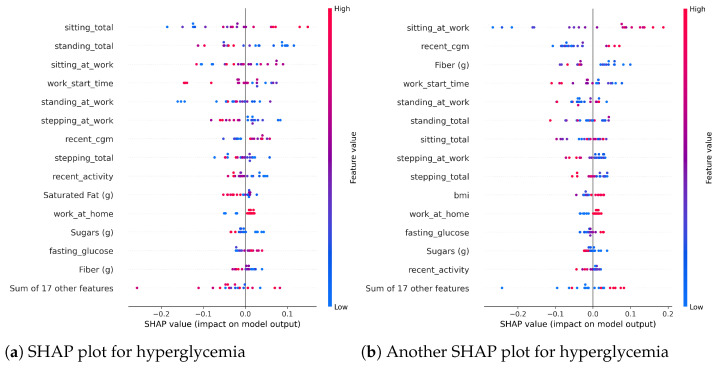

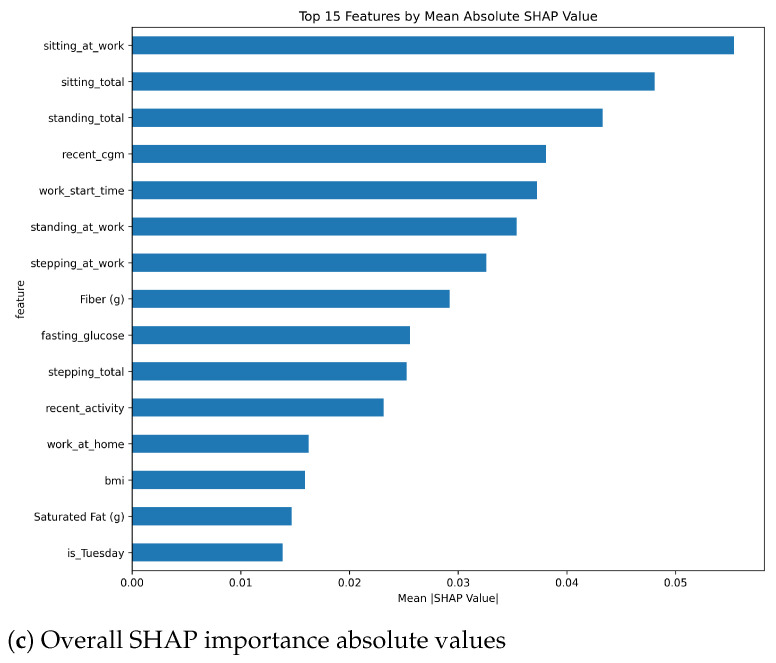
Two SHAP plots and the overall SHAP importance absolute values calculated from 5 randomized experimental trials. The beeswarm plots in (**a**,**b**) show how different feature values contribute toward hyperglycemia prediction. The absolute values in (**c**) show only the importance and not the direction, i.e., whether the feature contributes positively or negatively toward hyperglycemia. An increasing amount of fat and fiber for the same amount of carbs can slow down gastric emptying and glucose absorption [45,50]. Standing and sitting durations decrease and increase the likelihood of getting hyperglycemia, respectively, which is also consistent with the current clinical literature [46,47]. Therefore, the SHAP values shown in the subfigures are consistent with clinical understanding. The contribution of the amount of sugars shown in the plots may be counterintuitive, as the SHAP plots suggest a lower amount of sugars can contribute toward increasing the likelihood of hyperglycemia. We believe it may be an effect of other factors that are correlated with the amount of sugar consumed by most of our participants.

The SHAP (SHapley Additive exPlanations) analysis revealed key features influencing the hyperglycemia prediction model, as depicted in Figure 11. Figure 11a,b present SHAP beeswarm plots, where each dot corresponds to a feature’s contribution to individual predictions, with the x-axis representing the SHAP value (indicating positive or negative impact on the model output) and the y-axis listing features. Color coding (red for high values, blue for low) highlights the significant positive contributions of “sitting_at_work” and “standing_total” to the model output when their values are elevated. Figure 11c ranks the top 15 features by mean absolute SHAP values, identifying “sitting_at_work”, “standing_total”, and “sitting_total” as the most influential, followed by “recent_cgm” and “work_start_time”, underscoring their critical roles in predicting hyperglycemia risk. Moreover, the SHAP values provided for the predictions made by our system are consistent with the clinical understanding, as explained in Figure 11.

## 4. Discussion

### 4.1. Summary of the Results

We have presented an important problem of estimating the postprandial area under the curve (AUC), the maximum postprandial blood glucose level (MaxBGL), and detecting hyperglycemia. Based on our experiments, we found that Random Forest (RF) models outperform Multilayer Perceptrons (MLPs), ridge regression, XGBoost, and TabNet models in AUC prediction, as shown in Table 4. We also chose our features from five different feature sets, as illustrated in Table 1. Although our feature sets use 31 features as input to the model when choosing the set of ‘All’ features, all these features are derived from only four sources of data, which makes it easier to have the required data easily available when needed for the model.

Our LLM-based experiments suggested that Claude Opus 4 outperforms GPT 3.5 Turbo, GPT 4, Gemini 2.0 Flash 001, DeepSeek V3, and Grok 3 in AUC predictions. We also found that hybrid models outperform LLM-only models, whereas augmented training data with Gaussian noise further improves the performance of the hybrid models. However, fully trainable base regressor backbones of our GlucoLens system outperform both LLM-based and hybrid versions of the predictors for AUC prediction.

Finally, in hyperglycemia prediction, we find that GlucoLens with ensemble classifiers RF, XGB, and MLP outperforms the system with individual classifiers, achieving an accuracy of 79% and an F1 score of 0.749, as shown in Table 6, Table 7 and Table 8. Additionally, we empirically prove that the GlucoLens hyperglycemia prediction system monotonically improves its test set performance as we increase the size of the training data.

### 4.2. Different Interpretation of the Results

Our best-found NRMSE of 0.123 implies that the predicted AUC value was on average within a 12.3% error margin from the actual values of AUC. To interpret the performance in another way, we also explored the percentage of test cases falling within an error tolerance. From the actual AUC values and the predicted AUC values, the ratio of test cases within 5%, 10%, 15%, and 20% errors were calculated. Among the predictions made by our system, we have verified that 33% of the cases had an error of less than 5%, 61% of the cases had an error of less than 10%, 81% of the cases had an error of less than 15%, and 93% cases had an error of less than 20%. Thus only 19% of the cases had an error of more than 15%, and only 7% of the cases had an error over 20%. We believe that with more training data, our system will be able to predict most of the cases within a very small margin of error. The percentages of test cases within 5%, 10%, 15%, and 20% error margins for the XGBoost model were 30%, 55%, 78%, and 92%, respectively, all lower than our Random Forest model. Therefore, our Random Forest model outperforms XGBoost in this metric as well.

### 4.3. Alternative Feature Search Methods

We also explored another avenue of research to find possible feature sets other than the five options presented in Table 1. One way could be to perform an exhaustive search within the features. The problem with that approach would be the computational cost. As we have 31 features, we would have $2^{31}$ possibilities that would be infeasible to explore. However, we have performed an additional experiment to find effective feature subsets. Among all the models, our Random Forest (RF) was the best model so far with an NRMSE of 0.123. For finding alternative subsets, we limited the number of features by restricting the number of leaf nodes in RF models to 24, 48, and 96. The 48-leaf-node limit outperformed the others, achieving an NRMSE of 0.121, surpassing our previous best result of 0.123. This approach offers a more rational feature selection method, as limiting leaf nodes helps identify features with higher information gains, reduces overfitting, and improves generalization.

### 4.4. Reflection on LLM-Based Prediction

Our experimental results indicate that current large language models (LLMs) did not outperform classical machine learning approaches in predicting hyperglycemia. While LLMs offer promising generalization and reasoning capabilities, we believe they are not yet sufficiently optimized for accurate physiological prediction tasks such as postprandial glucose estimation. Despite their current limitations, the LLM-based interface we developed provides a flexible and scalable framework that can be reused or extended as more advanced LLMs become available. Our initial motivation for experimenting with multiple LLMs was to evaluate whether combining their outputs, through a hybrid or ensemble method, could improve prediction accuracy. However, our findings suggest that using the single best-performing LLM is more effective than relying on a mixture of models.

In practice, combining multiple LLMs adds marginal inference overhead as LLMs are not retrained or fine-tuned in this process. Furthermore, it does not create an additional burden on the end-user. Our future work will focus on optimizing LLMs with a manageable number of parameters for academic research and explore whether specialized LLMs and fine-tuned LLMs for lightweight, single-model inference is more practical.

Future work may revisit this approach as LLMs continue to evolve, especially with the emergence of models better aligned with domain-specific reasoning and capable of leveraging structured and temporal health data more effectively.

## 5. Limitations and Future Works

The proposed system addresses an important problem in metabolic health, namely glucose control in individuals with prediabetes. However, we also acknowledge several limitations. The LLM-based models did not outperform the classical models. However, we believe that future generations of the LLMs will perform better with the same interface. Secondly, because of a lack of existing research, our prompt design was based on simplicity and interpretability. In the future, we plan to explore different prompt designs and also fine-tune small language models for better results. Finally, the interventions provided by the counterfactual explanation tools will need to be evaluated by deploying such interventions in clinical trials. Additionally, we would like to design a digital twin solution to simulate the efficacy of the generated counterfactual interventions.

As a next step, we plan to explore the development of personalized glucose prediction models that account for individual variations in behavior and dietary habits. A potential approach involves a two-phase framework. In the initial training phase, each user would wear a continuous glucose monitor (CGM) for a period of 1–2 weeks, during which their food intake and daily activities are logged. These data would be used to train a personalized model capable of capturing the user’s unique glycemic response patterns. Once trained, the model would enter the deployment phase, where it could predict postprandial glucose levels and assess hyperglycemia risk without requiring ongoing CGM usage. Instead, predictions would rely solely on behavioral and dietary inputs, offering actionable insights and personalized lifestyle recommendations.

We also envision integrating automated tools that can extract caloric and macronutrient information from food photos or meal descriptions, which would streamline data collection and reduce user burden. Additionally, future work will address ethical and data privacy concerns associated with using personal health data, with an emphasis on secure data handling and privacy-preserving model deployment strategies.

## 6. Conclusions

We developed GlucoLens, an interpretable ML system for estimating postprandial blood glucose parameters by analyzing features associated with diet and physical activity. We believe that the developed tools will be helpful to people diagnosed with or at risk for diabetes to better regulate their condition and avoid adverse health outcomes such as long-term chronic complications. GlucoLens is a multi-sensor system that leverages advanced data processing to generate multimodal feature sets and applies machine learning regressors, classifiers, and large language models to predict and explain hyperglycemia. Based on the experiments with different variations in models and feature sets, the GlucoLens AUC prediction system, in its best configuration, outperforms the comparison models by a margin of 10% to 27%. Moreover, GlucoLens achieves a 79% accuracy and an F1 score of 0.749 on hyperglycemia prediction. Finally, it recommends interventions to avoid hyperglycemia using diverse counterfactual explanations. Based on counterfactual explanations and SHAP explanations, we showed that a regulated diet and higher physical activity can avoid hyperglycemia.

## Figures and Tables

**Table 1:** A comparison of the five different feature sets used in this study. The checkboxes indicate whether a feature is present in a specific feature set. GL = glycemic load of the meal. Self = activity scores calculated by self-reported activity duration in work logs. Macro = macronutrients that are used to calculate glycemic load: net carb, fat, protein, and carb. Sensor = activity metrics from the activPAL sensor: duration of sitting, standing, and stepping activities of the day before lunch and the same metrics from the start of working to just before lunch of the same day.

No.	Feature Name/Shorthand	Sensor + GL	Sensor + Macro	Self + GL	Self + Macro	All	Description
1	Fasting glucose	⊠	⊠	⊠	⊠	⊠	Minimum CGM reading between 6 AM and 10 AM. (mg/dL).
2	Recent CGM	⊠	⊠	⊠	⊠	⊠	Mean glucose concentration from 12 AM to 8 AM (mg/dL)
3	Lunch time	⊠	⊠	⊠	⊠	⊠	Time when lunch was consumed (HH:MM).
4	Work from home	⊠	⊠	⊠	⊠	⊠	Binary flag indicating work from home (0 or 1).
5	BMI	⊠	⊠	⊠	⊠	⊠	Body Mass Index (kg/m^2^).
6	Calories	⊠	⊠	⊠	⊠	⊠	Total meal calories (kcal).
7	Calories from fat	⊠	⊠	⊠	⊠	⊠	Caloric contribution from fat (kcal).
8	Saturated fat	⊠	⊠	⊠	⊠	⊠	Saturated fat content of meal (g).
9	Trans fat	⊠	⊠	⊠	⊠	⊠	Trans fat content of meal (g).
10	Cholesterol	⊠	⊠	⊠	⊠	⊠	Cholesterol in the meal (mg).
11	Sodium	⊠	⊠	⊠	⊠	⊠	Sodium intake from the meal (mg).
12	Total carbs	⊠	⊠	⊠	⊠	⊠	Total carbohydrate amount (g).
13	Sugar	⊠	⊠	⊠	⊠	⊠	Sugar content in the meal (g).
14	Work start time	⊠	⊠	⊠	⊠	⊠	Time when the user started working (HH:MM).
15	Day of the week	⊠	⊠	⊠	⊠	⊠	Categorical variable indicating weekday (Mon–Sun).
16	activPAL	⊠	⊠	⃞	⃞	⊠	Sitting, standing, and stepping durations for the same day until the time of lunch as well as those durations at work for that day before lunch (seconds).
17	Self-reported activity	⃞	⃞	⊠	⊠	⊠	Sitting, standing, and stepping durations manually reported (percentages of the work duration for each day).
18	GL	⊠	⃞	⊠	⃞	⊠	Glycemic load of the meal (unitless index).
19	Net carbs	⃞	⊠	⃞	⊠	⊠	Total carbs minus fiber (g).
20	Fat	⃞	⊠	⃞	⊠	⊠	Total fat content in the meal (g).
21	Protein	⃞	⊠	⃞	⊠	⊠	Protein content of the meal (g).
22	Fiber	⃞	⊠	⃞	⊠	⊠	Dietary fiber amount in the meal (g).
23	AUC	⃞	⃞	⃞	⃞	⃞	Outcome variable. 3-h postprandial area under the curve (mg/dL · h). In this study, we used the absolute AUC value without normalizing.

**Table 2:** An overview of the different backbones and their hyperparameters for the regression problem. Explanations of the feature sets can be found in Table 1. MLP = Multilayer Perceptron. RF = Random Forest. Ridge = Ridge Regression. $n_{est}$ = number of estimators in random forest. Meanings of the outcomes AUC and MaxBGL can be found in Figure 1. Gly_Hybrid = best performing regressor (RF) with support of LLM-based predictions, Gly_Hybrid_v2 is RF with LLM-based predictions but with only the best LLM (Claude Opus 4). Gly_Max is Gly_Hybrid_v2 with augmented training data with Gaussian noise. Augmentation was performed only on the training data and not on the test data.

Target outcomes	AUC, MaxBGL, Hyperglycemia
Feature	Sensor + Macro, Self + Macro,
sets	Sensor + GL, Self + GL, All
	RF, Ridge, MLP, XGBoost, TabNet, GPT-3.5, GPT-4, Mistral Large,
Predictors	Gemini Flash 2.0, Claude Opus 4, Grok 3, Deepseek V3, Gly_Hybrid,
	Gly_Hybrid_v2, Gly_Max, Hybrid Predictors for Classification
	(RF+MLP, RF+XGB, XGB+MLP, RF+XGB+MLP).
Ridge variations	α∈{1,0.1,0.01}
RF variations	$n_{est}\in \left\{10,50,100\right\}$
MLP variations	13 variations; see Table 3

**Table 3:** Variations of the multilayer perceptron (MLP regressor). Different variations have been tested by varying the depth and size of each layer.

Variation	# Hidden	# Nodes in
No.	Layers	Hidden Layers
1	3	(20, 10, 5)
2	4	(40, 20, 10, 5)
3	4	(60, 30, 15, 7)
4	5	(80, 40, 20, 10, 5)
5	5	(100, 50, 25, 12, 6)
6	5	(120, 60, 30, 15, 7)
7	5	(140, 70, 35, 17, 8)
8	5	(160, 80, 40, 20, 10)
9	8	(80, 40, 20, 20, 20, 20, 10, 5)
10	8	(100, 50, 25, 25, 25, 25, 12, 6)
11	8	(120, 60, 30, 30, 30, 30, 15, 7)
12	8	(140, 70, 35, 35, 35, 35, 17, 8)
13	8	(160, 80, 40, 40, 40, 40, 20, 10)

**Table 4:** Normalized Root Mean Squared Errors (NRMSEs) of our GlucoLens models (RF, Ridge, MLP, XGBoost, TabNet) for different feature sets in the prediction of postprandial AUC. Explanations of the feature sets can be found in Table 1. Boldfaced values represent the best results for the corresponding feature sets.

Feature Set	RF	Ridge	MLP	XGBoost	TabNet
Sensor + GL	**0.125**	0.139	0.169	0.137	0.160
Sensor + Macro	**0.123**	0.142	0.172	0.139	0.147
Self + GL	0.142	**0.139**	0.178	0.152	0.154
Self + Macro	**0.139**	0.142	0.172	0.149	0.151
All	**0.123**	0.140	0.176	0.137	0.151

**Table 5:** AUC NRMSE results of different variations in our solution. Gly_Base = GlucoLens regressor with no LLM, Gly_LLM = LLM only prediction (zero-shot) after multimodal data processing by GlucoLens. The hybrid predictors are explained in Table 2. The best result is bolded.

Backbone	Gly_Base	Gly_LLM	Gly_Hybrid	Gly_Hybrid_v2	Gly_Max
RF	**0.123**	0.281	0.241	0.238	0.226
XGBoost	0.137		0.236	0.242	0.259

**Table 6:** GlucoLens system’s classification results with different pure and hybrid backbones for hyperglycemia detection on the 87% training and 13% test split. All metrics are averages over 100 repetitions with different random seeds. Best results and the best configuration are bolded.

Classifier	Accuracy	Precision	Recall	F1
RF	0.698	0.737	0.699	0.685
XGB	0.685	0.720	0.692	0.682
MLP	0.620	0.626	0.620	0.589
RF+XGB	0.695	0.730	0.695	0.683
RF+MLP	0.668	0.700	0.668	0.650
XGB+MLP	0.687	0.712	0.687	0.672
**RF+XGB+MLP**	**0.712**	**0.740**	**0.712**	**0.702**

**Table 7:** Results of the GlucoLens hyperglycemia detection system with RF+XGB+MLP-based hybrid backbone as we increase the training data size. All metrics are averages over 100 repetitions with different random seeds. An improvement in the performance metrics can be observed except for the last row, when only 1% of the dataset is withheld for testing. In that case, the evaluation in any trial is vulnerable to producing 0 values for precision, recall, or F1 score, as there is only 1 example from each class in the test set. Eventually, it affects the overall average of those metrics. Best results and the best configuration are bolded.

Size of Training Set	Accuracy	Precision	Recall	F1
70% training, 30% test	0.674	0.706	0.674	0.660
80% training, 20% test	0.660	0.729	0.702	0.690
87% training, 13% test	0.712	0.740	0.712	0.702
90% training, 10% test	0.717	0.744	0.717	0.705
**95% training, 5% test**	**0.733**	**0.751**	**0.733**	**0.716**
99% training, 1% test	0.730	0.625	0.730	0.660

**Table 8:** Comparison of hyperglycemia prediction performance of RF+XGB+MLP hybrid classifier with and without data augmentation. A 10-fold cross-validation was used for classification. No balancing or augmentation was performed on the test set in either case. Best average results are bolded.

	Accuracy	Precision	Recall	F1 Score
Without Augmentation				
Trial 1	0.786	0.749	0.749	0.749
Trial 2	0.755	0.714	0.721	0.717
Trial 3	0.748	0.705	0.705	0.705
Average	0.763	0.723	0.725	0.724
With Augmentation				
Trial 1	0.774	0.735	0.723	0.728
Trial 2	0.786	0.749	0.749	0.749
Trial 3	0.811	0.783	0.762	0.771
Average	**0.790**	**0.756**	**0.745**	**0.749**

**Table 9:** Leave-one-subject-out evaluation with different variations for generalizability testing.

Variation	Accuracy	Precision	Recall	F1 Score
No personalization or augmentation	0.600	0.572	0.568	0.568
With augmentation	0.630	0.601	0.589	0.589
With personalization	0.648	0.611	0.615	0.611
With personalization and augmentation	0.690	0.654	0.651	0.651

**Table 10:** Leave-one-out analysis results.

Trial	Accuracy	Precision	Recall	F1 Score
Trial 1	0.755	0.712	0.710	0.711
Trial 2	0.774	0.735	0.735	0.735
Trial 3	0.786	0.752	0.772	0.759
Average	0.772	0.733	0.739	0.735

**Table 11:** Summary of the evaluation metrics of the counterfactual explanations. Here, we can see that all the generated counterfactuals were valid, i.e., the generated counterfactuals belonged to the opposite class. The normalized distance was 2.258 units from the original example. Average normalized diversity was 3.945 units, i.e., the counterfactuals of the same examples were diverse enough to provide the user with different options. Finally, on average, only 2 features need to be changed in the counterfactuals.

Metric	Value
Average validity	1.000
Average diversity	3.945
Average normalized distance	2.258
Average features changed	2.000

## Data Availability

The data is currently not publicly available. Only a few of the research team members, whose role in this study required access to the identifiable information of the participants, were given such access, adhering to the protocol approved by the IRB. Rest of the team members had access to only the anonymized data. All the contributors of this research were added to the IRB record as team members.
